# Causal pathways linking environmental change with health behaviour change: Natural experimental study of new transport infrastructure and cycling to work

**DOI:** 10.1016/j.ypmed.2016.02.042

**Published:** 2016-06

**Authors:** R.G. Prins, J. Panter, E. Heinen, S.J. Griffin, D.B. Ogilvie

**Affiliations:** a MRC Epidemiology Unit and UKCRC Centre for Diet and Activity Research (CEDAR), School of Clinical Medicine, University of Cambridge, Cambridge, United Kingdom; b Primary Care Unit, Department of Public Health and Primary Care, University of Cambridge, Cambridge, United Kingdom

**Keywords:** Natural experiment, Environment design, Effectiveness, Psychosocial factors, Physical activity, Walking, Cycling

## Abstract

**Background:**

Mechanisms linking changes to the environment with changes in physical activity are poorly understood. Insights into mechanisms of interventions can help strengthen causal attribution and improve understanding of divergent response patterns. We examined the causal pathways linking exposure to new transport infrastructure with changes in cycling to work.

**Methods:**

We used baseline (2009) and follow-up (2012) data (*N* = 469) from the Commuting and Health in Cambridge natural experimental study (Cambridge, UK). Exposure to new infrastructure in the form of the Cambridgeshire Guided Busway was defined using residential proximity. Mediators studied were changes in perceptions of the route to work, theory of planned behaviour constructs and self-reported use of the new infrastructure. Outcomes were modelled as an increase, decrease or no change in weekly cycle commuting time. We used regression analyses to identify combinations of mediators forming potential pathways between exposure and outcome. We then tested these pathways in a path model and stratified analyses by baseline level of active commuting.

**Results:**

We identified changes in perceptions of the route to work, and use of the cycle path, as potential mediators. Of these potential mediators, only use of the path significantly explained (85%) the effect of the infrastructure in increasing cycling. Path use also explained a decrease in cycling among more active commuters.

**Conclusion:**

The findings strengthen the causal argument that changing the environment led to changes in health-related behaviour via use of the new infrastructure, but also show how some commuters may have spent less time cycling as a result.

## Highlights

• Environmental perceptions did not explain effects of new infrastructure on cycling.
• Motivational factors did not explain effects of new infrastructure on cycling.
• Eighty-five percent of the effect on increasing cycling was explained by use of the infrastructure.
• These results strengthen quasi-experimental causal attribution and understanding.

## 1 Introduction

A shift towards more active travel could bring population health and societal benefits (Beaglehole et al., 2011, Douglas et al., 2011, Statistics Netherlands, 2013). For example, higher levels of walking and cycling are positively associated with overall physical activity levels (Sahlqvist et al., 2012) and better health outcomes and may result in reductions in health care costs (Audrey et al., 2014, Jarrett et al., 2012). The prevalence of active commuting differs widely between countries. In the UK, Switzerland, Canada and the USA, fewer than 15% of adults use active modes of travel to work, whereas in countries such as China, the Netherlands, France and Germany, more than 30% do so (Hallal et al., 2012).

Environmental interventions have the potential to achieve sustained shifts in population travel behaviour, but there is scientific uncertainty about their effects (Fraser and Lock, 2011, Health Council of the Netherlands, 2010, National Institute for Health and Clinical Excellence, 2008, Pucher et al., 2010). This reflects not only the lack of evaluation studies but also the challenges involved in attributing the observed outcomes to the interventions studied. For example, evaluations of effectiveness may be confounded if intervention and control areas differ in broader contextual factors and randomisation is unfeasible (Craig et al., 2012). There may therefore be other explanations for an apparent “intervention effect” (Keall et al., 2015). To exclude the possibility that such effects are non-causal, it is important to understand the pathways by which an intervention brings about its effects (Rothman and Greenland, 2005, Rychetnik et al., 2002, Victora et al., 2004, Zabaleta-del-Olmo et al., 2015). Together with estimating the causal effect, elucidating these mechanisms can help support causal inference.

Insights into such mechanisms may also help improve the design, targeting and implementation of interventions. Socio-ecological frameworks hypothesise that cognitions, including perceptions of the environment, may lie on the pathways linking environment and behaviour (Alfonzo, 2005, Ogilvie et al., 2011, Sallis et al., 2006). To date, the evidence about such pathways comes mostly from cross-sectional studies. Some of these have found that environmental perceptions mediate the pathway between environmental factors and active travel (Van Dyck et al., 2013). Others have found that attitudes and intention to walk mediate the associations between perceptions of aesthetics or land-use mix and walking behaviour (Rhodes et al., 2006, Rhodes et al., 2007). Whilst such cross-sectional studies provide some preliminary, non-causal evidential support for pathways by which environments may influence active travel, evidence from intervention studies is lacking (McCormack and Shiell, 2011). As far as we are aware, only one intervention study has investigated the causal pathways between environmental change and physical activity behaviour change. It showed that new walking and cycling routes to increase the connectivity of local areas were associated with changes in environmental perceptions. However, the effect of the new routes on walking and cycling behaviour was mainly explained by use of the new infrastructure, not by changes in perceptions (Panter and Ogilvie, 2015).

In this study, we aimed to evaluate the causal mechanisms linking exposure to new transport infrastructure with changes in commuter travel behaviour. We focused on cycling, because the main outcome evaluation found positive effects on cycle commuting — with a relative risk ratio for an increase in cycling of 1.34 (95% CI 1.03 to 1.76) — but not on walking (Panter et al., 2016). However, we also report results for walking in an appendix.

## 2 Methods

### 2.1 Intervention, study design and participants

The intervention and study protocol are described in full elsewhere (Ogilvie et al., 2010). Briefly, the Cambridgeshire Guided Busway (hereafter referred to as the busway) links towns and villages to the north-west of Cambridge (UK) with the Cambridge Science Park, the city centre and the Cambridge Biomedical Campus. Buses run, largely, on tracks separated from other traffic to the north and south of the city centre; between these sections, they use the regular street network. The busway includes two new park-and-ride sites, and the southern section terminates at an existing park-and-ride site. A path for walking and cycling runs alongside the guideway. Construction began in spring 2007, and the busway was opened in summer 2011.

In this analysis, we used baseline (2009) and 3-year follow-up (2012) data, augmented by information on use of the infrastructure collected at intermediate time points in 2010 and 2011. We recruited participants through workplaces using a variety of methods including emails, leaflets and recruitment stands. At each wave of data collection, participants were entered in a prize draw to win one of eight £50 gift vouchers. Adults (≥ 16 years), who lived within 30 km of the city centre and travelled to workplaces in Cambridge to be served by the busway, were eligible for inclusion. Of the 2163 people who expressed an interest in taking part, 1582 met the inclusion criteria and were sent a baseline postal questionnaire. Of these, 1164 (74%) returned the baseline questionnaire and a consent form. Follow-up postal questionnaire data were returned by 469 (40%) of the baseline participants. Those who provided baseline and follow-up data tended to be older (mean age = 44.3 vs. 40.9 years, *p* = 0.001) and more likely to be homeowners (78% vs. 69%, *p* = 0.001) than participants who did not complete follow-up (Heinen et al., 2015b). Census data from Cambridge city and surrounding areas suggested that the sample had a higher proportion of women, older adults, those who had a degree and those who owned their home than the local population (Panter et al., 2016).

The Hertfordshire Research Ethics Committee approved the study and baseline data collection (reference number: 08/H0311/208), and the Cambridge Psychology Research Ethics Committee approved the follow-up data collection (reference number: 2014.14). All participants gave written informed consent.

## 3 Measures

### 3.1 Cycling on the commute

At baseline and follow-up, participants reported all their commuting journeys and the modes of transport used over the past 7 days. Participants who cycled any part of their journeys to and from work were asked to report the average time cycled per trip. We calculated total weekly cycle commuting time by multiplying the number of trips involving cycling with the average duration of cycling per trip. This measure has been shown to have acceptable criterion validity, with only a small mean overestimation compared to objectively-derived estimates (Panter et al., 2014).

We calculated change in time spent cycling by subtracting the weekly cycle commuting time at baseline from the weekly cycle commuting time at follow-up. This variable was skewed and was therefore categorised as either a decrease, no change (reference category) or an increase in weekly cycle commuting time. We generated a similar measure for walking (Appendix A).

### 3.2 Exposure to the busway

We calculated exposure to the intervention using the road network distance from each participant's home to the nearest access point to the busway. This could be a bus stop or an access point on the path because changes in commuting behaviour could reflect walking or cycling either as single modes of transport or in combination with public transport. The distance was calculated in ArcGIS 9.1, using the Ordnance Survey Integrated Transport Network and the Open Street Map databases. Analyses indicated that use of the busway decreased with distance in a non-linear manner (Heinen et al., 2015a). We therefore applied a square root transformation to the negative of the distance and used this to represent exposure to the intervention (Heinen et al., 2015a, Heinen et al., 2015b, Panter et al., 2016).

### 3.3 Mediators

We measured theory of planned behaviour (TPB) constructs (Hardeman et al., 2009) by questionnaire at baseline and follow-up, using a five-point scale from “strongly disagree” (1) to “strongly agree” (5). As it was considered unfeasible to repeat the TPB measures in respect of all modes of transport, they were asked in relation to car use because the overarching aim of the intervention was to promote the use of alternatives to the car. Each TPB construct was measured with two items. Attitude was measured with the items ‘Overall, it would be good to use a car’ and `It would be pleasant to use a car’; perceived behavioural control with ‘It would be easy for me to use a car’ and ‘I would be able to use a car’; subjective norm with ‘Most people who are important to me would support me using a car’ and ‘Most people who are important to me think I should use a car’; and intention with ‘I intend to use a car’ and ‘I am likely to use a car’.

Each pair of items showed a Cronbach's alpha of at least 0.8 at each time point. A summary measure for each construct at each time point was therefore calculated by taking the mean of the two items.

Seven items measured perceptions of the environment en route to work by asking participants to indicate their level of agreement, on the same scale, with the statements ‘It is pleasant to walk’, ‘The roads are dangerous for cyclists’, ‘There is convenient public transport’, ‘There are convenient routes for cycling’, ‘There is little traffic’, ‘There are no convenient routes for walking’ and ‘It is safe to cross the road’.

Change scores for the TPB constructs and perceived environmental factors were created by subtracting the baseline values from the follow-up values.

use of the intervention was assessed at follow-up waves in 2010, 2011 and 2012 with the item “Have you walked or cycled along any part of the footpath or cycle path beside the guided busway?”. The response categories were “Yes, I have walked beside the Busway”, “Yes, I have cycled beside the Busway” and “No, I have not walked or cycled along the paths beside the busway at all”. A dichotomous summary measure was created to indicate whether a participant had ever reported cycling beside the busway, hereafter referred to as “self-reported use”. We generated a similar measure for walking (Appendix A). A detailed description of the items is given in Appendix B.

### 3.4 Covariates

Covariates were demographic (gender, age, presence of children under 16 years in the household), socio-economic (education, change in car ownership, change in home ownership) and environmental (urban–rural status, change in the availability of car parking at work, moving home or workplace) characteristics and weekly cycle commuting time at baseline. All covariates were derived from the baseline questionnaire or from changes between baseline and follow-up questionnaire responses as appropriate.

## 4 Analyses

Descriptive statistics were calculated for the covariates and changes in weekly cycle commuting time.

**Fig. 1:**
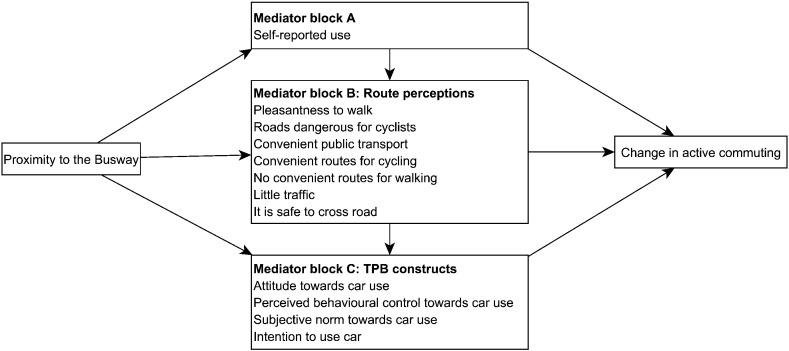
Conceptual model. TPB: theory of planned behaviour.

Our aim was to go beyond single mediator models by simultaneously evaluating multiple pathways consisting of one or more mediating variables by which proximity to the busway might affect commuter cycling behaviour. Our conceptual model, based on socio-ecological frameworks, (Kremers et al., 2006, Ogilvie et al., 2011) involved three blocks of hypothesised mediators: (a) self-reported use of the busway, (b) changes in route perceptions and (c) changes in TPB constructs (Fig. 1).

We took a two-step approach to our analyses. In the first step, we aimed to limit the number of pathways to be tested, by deriving a refined model containing the pathways that were *most likely*, based on a procedure developed previously (Panter and Ogilvie, 2015). In the second step, we tested the significance of these ‘plausible’ pathways.

In the first step, we first tested *all* of the associations (adjusted for covariates) between *all* the variables in the model (Fig. 1) in STATA 13. We used linear, logistic or multinomial regression models as appropriate for each association tested. The model was refined by excluding paths between variables that were not statistically significant (*p* < 0.05). We then excluded mediators that were associated only with a mediator from the same block and then excluded paths that contained more than two mediators from the same block. This limited the length of the pathways to be tested. In line with our conceptual model, we only considered combinations of mediators that were ordered according to Fig. 1 (i.e. in which use precedes changes in route perceptions, and changes in route perceptions precede changes in TPB constructs). Participants were included in these analyses if they provided complete data on the outcome, the potential mediators and the covariates.

The statistical significance of each path identified was tested in the second step with path regression models in MPlus 7.1, using full-information maximum-likelihood estimation with 1000 iterations and adjusting for covariates. Although this procedure is efficient in handling missing data, MPlus dropped missing data on the covariates from the analyses. We further explored whether the paths differed between those with lower and higher levels of active commuting at baseline. Participants were categorised using the median split of weekly active commuting time (the sum of time spent walking and cycling for commuting) and all the previous steps were repeated in each stratum.

The same analyses were conducted using weekly walking commuting time as the outcome (Appendix A).

## 5 Results

### 5.1 Sample characteristics

The study samples used in both analytical steps are described in Table 1. A total of 414 participants were in the sample used to identify the potential pathways (step 1) and 456 participants were included in the path analyses (step 2). We did not observe any statistically significant differences either between these samples, or between either sample and the whole sample providing baseline and follow-up data.

### 5.2 Analysis step 1: Identification of plausible pathways

**Fig. 2:**
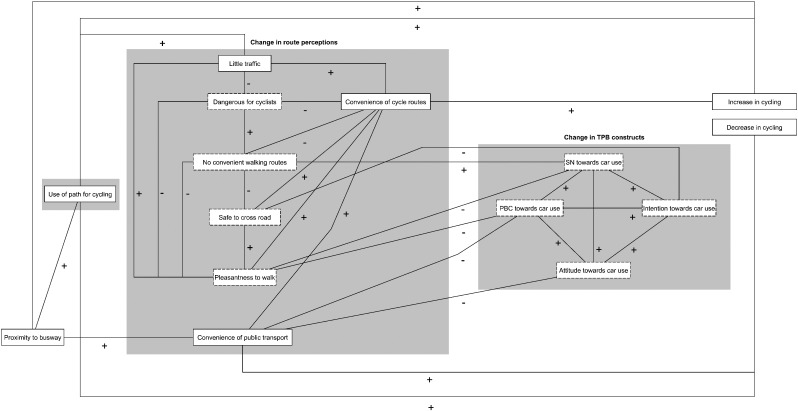
First refined path model linking exposure to the intervention with changes in cycle commuting time. All associations shown are statistically significant (*p* < 0.05) and either positive (+) or negative (−); dotted boxes denote potential mediators that violated the inclusion criteria for pathways to be tested (see Methods); PBC: perceived behavioural control; SN: subjective norm.

Figure 2 shows the direction (positive or negative) of the statistically significant results (*p* < 0.05) of the regression analyses adjusted for all covariates. The dotted boxes represent variables that were excluded in the process of refining the conceptual model. For example, “Roads dangerous for cyclists” was associated only with mediators from the same block and was therefore removed from the model.

#### 5.2.1 Pathways towards an increase in cycling

**Fig. 3:**
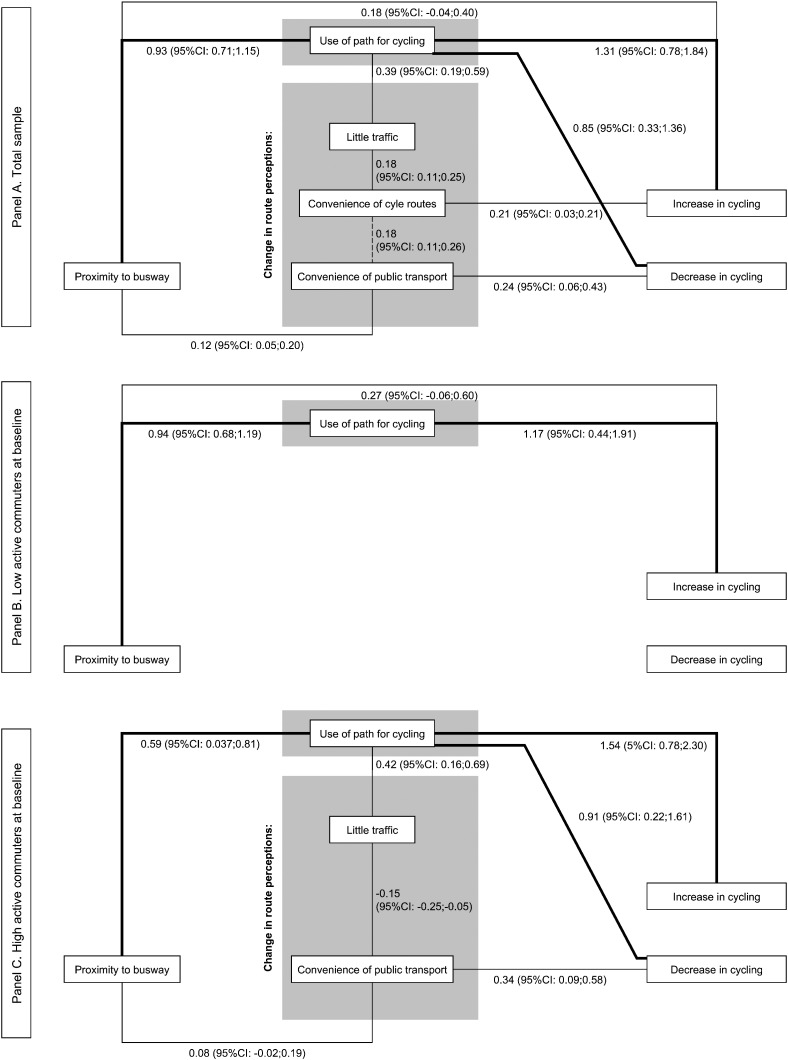
Second (reduced) refined path models for whole sample and subsamples with lower and higher levels of active commuting at baseline Values represent regression coefficients (95% confidence intervals) from the path analysis; bold lines denote statistically significant *pathways*.

We identified one direct and three indirect pathways linking the busway with an *increase in* cycling (Fig. 3A). In the direct pathway, exposure to the busway was positively associated with an increase in cycling (Path 1.0). In the first indirect pathway, exposure to the busway was positively associated with use of the busway, which in turn was also positively associated with an increase in cycling (Path 1.1). In the second indirect pathway, use was associated with increased perceptions of little traffic, which was associated with increased perceptions of convenience of cycle routes, which was associated with an increase in cycling (Path 1.2). In the third indirect pathway, proximity to the busway was positively associated with perceptions of more convenient public transport, which was associated with increased perceptions of convenience of cycle routes, which was positively associated with an increase in cycling (Path 1.3).

#### 5.2.2 Pathways towards a decrease in cycling

Two indirect pathways linking the busway with a decrease in cycling were identified (Fig. 3). The first pathway showed that people living closer to the busway were more likely to use it, and that those who used it were more likely to reduce their cycling (Path 2.1). The second pathway showed that people living closer to the busway were more likely to perceive more convenient public transport, and that those who perceived more convenient transport were more likely to reduce their cycling (Path 2.2).

For walking, one pathway linking the busway with an increase in walking and four pathways linking it with a decrease in walking were identified (Appendix A).

### 5.3 Analysis step 2: testing the path models

The indirect paths via use of the busway to increased and decreased cycling were both statistically significant (*B* = 1.22, 95% CI = 0.64–1.79 and *B* = 0.79, 95% CI = 0.27–1.30, respectively; Table 2). None of the other indirect pathways towards a decrease in cycling, and none of the pathways towards a change in walking, were statistically significant (Table 2 and Appendix A).

In the stratified analysis for cycling, six plausible pathways were identified (Fig. 3 and Table 3). A statistically significant indirect pathway via use of the busway towards an increase in cycling was identified among those with lower levels of active commuting at baseline (*B* = 1.10, 95% CI = 0.35–1.85). Among those with higher levels of active commuting, statistically significant indirect pathways were identified via use of the busway towards both an increase (*B* = 0.90, 95% CI = 0.35–1.46) and a decrease (*B* = 0.54, 95% CI = 0.08–1.46) in cycling.

For walking, no pathways were identified for the group with lower levels of active commuting, and no statistically significant pathways were identified among those with higher levels of active commuting (Appendix A).

## 6 Discussion

### 6.1 Main findings

We aimed to understand the causal mechanisms through which proximity to new transport infrastructure resulted in changes in active travel on the commute. Having previously found no evidence for an overall effect of the busway on walking, we focussed our analyses on cycling. While we had observed an effect of proximity to the busway on cycle commuting, (Panter et al., 2016) such an association could have been confounded by other explanations (Keall et al., 2015). We identified plausible pathways by which proximity to the busway might influence time spent cycling, through use of the new infrastructure and changes in perceived characteristics of the route to work. However, only use of the busway for cycling explained the positive overall effect of the busway on cycle commuting reported previously. Changes in perceptions of the route to work did not explain these overall effects.

### 6.2 Strengths and limitations

This study is among the first to have examined the mechanisms through which environmental changes might lead to changes in physical activity behaviour in populations. In contrast to almost all previous research in this area, we investigated multiple mediators and path models using a longitudinal data set purposely collected to evaluate and understand the effects of an environmental intervention. This enabled us to advance the investigation beyond single mediator models and compare multiple pathways simultaneously.

In interpreting the findings, some limitations need to be taken into account. First, the TPB constructs were framed in terms of car use, whereas aligning them with the specific behaviours (Ajzen, 1991) and environmental factors (Prins et al., 2011) under investigation might have created stronger links between intervention and outcome. This would have resulted in a higher probability of detecting statistically significant mechanisms. Second, the pathways via cognitive factors involved more intermediate variables than the simpler pathway via use of the intervention. In combination with the comparatively weak associations between some variables, this may have weakened the overall pathways. Studies with greater statistical power are more likely to detect statistically significant mechanisms of this kind. However, our findings suggest that their relative contribution to the overall intervention effect is likely to be small.

### 6.3 Interpretation

Although it is important for causal inference to understand the pathways by which an environmental intervention brings about its effects (Rothman and Greenland, 2005, Rychetnik et al., 2002, Victora et al., 2004), there is a lack of research on such pathways from intervention studies. We are only just beginning to understand the pathways by which environmental changes may bring about changes in physical activity behaviours. According to socio-ecological models environmental factors influence behaviour through environmental perceptions and other cognitive factors (Alfonzo, 2005, Kremers et al., 2006, Sallis et al., 2006), for which cross-sectional studies provide some (albeit non-causal) supporting evidence (Rhodes et al., 2006, Rhodes et al., 2007, Van Dyck et al., 2013). However, in our analysis, environmental perceptions and constructs of the TPB did not appear to play a large role in explaining changes in active commuting. This is consistent with another study in which it was also found that perceived environmental factors were on pathways between exposure to changes in infrastructure and changes in active travel, but use of the infrastructure was the most important explanatory factor (Panter and Ogilvie, 2015). The fact that cognitive factors did not contribute to the effect on cycling for the commute may be due to the aforementioned limitations of this study. However, it is also likely that alternative, unmeasured mediators – for example, relating to travel time budgets (Ahmed and Stopher, 2014), or more unconscious pathways – may explain part of the intervention effect (Panter and Ogilvie, 2015). These should be investigated in future studies, probably by using mixture of qualitative and quantitative methods.

An apparently counterintuitive finding was that among highly active commuters at baseline, use of the busway for cycling explained both an increase and a decrease in cycling over time. There are at least two potential explanations for this finding. First, we identified a pathway from exposure to the busway, via more favourable perceptions of the environment, to a decrease in cycling. Although not statistically significant in the final analysis, this raises the possibility that providing a high-quality bus service, aimed specifically at commuters, offered a more attractive alternative than cycling for some commuters who p not to use the car, and therefore cycled before. Second, we used a measure of self-reported changes in the time spent cycling, and the new infrastructure may have reduced the actual or perceived cycling time for some commuters. Although exposure to the busway did not significantly shorten the route to work in the overall sample (Heinen et al., 2015a), for some commuters it may have provided a more efficient cycling route, with fewer junctions and consequently higher speeds. Consequences of this kind may explain why we observed divergent effects on cycle commuting time in our sample.

To conclude, we found that exposure to the intervention led to an overall increase in the time spent cycling on the commute, mainly through use of the new infrastructure for cycling. This increases the likelihood that the observed effect was truly causally associated with the intervention. But these results also suggest that mediators other than motivations or environmental perceptions may help explain how environments influence behaviour and why environmental adaptations are used or not used, and these should be explored in future studies. The novel approach we have developed and demonstrated to do this is also potentially transferable to studying mechanisms by which other types of population health interventions bring about their effects.

The following are the supplementary data related to this article.

Appendix A Pathways between exposure and change in walking on the commute Appendix A.

Appendix B Description of mediators Appendix B.

## Conflict of interest

The authors declare there is no conflict of interest.

## Transparency document

Transparency document. Image 1

## Figures and Tables

**Table 1:** Description of samples used to identify and test plausible pathways.

	**Sample used to identify plausible pathways (*N* = 414)**	**Sample used to test plausible pathways (*N* = 456)**
	**%/mean (SD)**	**%/mean (SD)**
Age	43.9 (10.8)	44.4 (11.0)
Gender		
Female	66.4%	66.2%
Male	33.6%	33.8%
Urbanicity		
Urban	65.9%	66.9%
Rural	34.1%	33.1%
Child		
No children	66.9%	68.2%
At least one child	33.1%	31.8%
Education		
Lower than degree level	25.4%	25.7%
Degree level	74.6%	74.3%
Car parking at work		
No	30.7%	30.7%
Yes, free	37.2%	37.1%
Yes, paid for	32.1%	32.2%
Home ownership		
Does not own a home	22.2%	21.7%
Owns a home	77.8%	78.3%
Car ownership		
Does not own a car	12.1%	11.4%
Owns at least one car	87.9%	88.6%
Baseline cycling (minutes per week)	93.6 (118.5)	92.8 (117.2)
Change in weekly cycling time		
Percentage increasing	23.2%	22.8%
Minutes increased among increasers	85.4 (71.8)	87.2 (74.9)
Percentage decreasing	31.6%	31.8%
Minutes decreased among decreasers	− 84.7 (65.5)	− 86.5 (68.3)

SD = standard deviation.

**Table 2:** Contribution of pathways in explaining the relation between exposure to the intervention and changes in cycle commuting time.

**Path**	***B* (95% CI)**	**% effect explained**
*Outcome*: *increase in cycling*		
Direct (Path 1.0)	0.18 (− 0.04, 0.40)	12.9%
Via use of path only (Path 1.1)	**1.22** (0.64, 1.79)	85.8%
Via use of path, little traffic and convenient cycle routes (Path 1.2)	0.01 (− 0.00, 0.03)	1.0%
Via convenient public transport and convenient cycle routes (Path 1.3)	0.01 (− 0.00, 0.01)	0.3%
**Total**	**1.42** (0.86, 1.97)	100%
		
*Outcome*: *decrease in cycling*		
Via use of path only (Path 2.1)	**0.79** (0.27, 1.30)	96.3%
Via convenient public transport only (Path 2.2)	0.03 (0.00, 0.06)	3.7%
**Total**	**0.81** (0.20, 1.33)	100%

*N* = 456. Bold figures are statistically significant values (*p* < 0.05); analyses were adjusted for covariates, based on full-information maximum-likelihood with 1000 iterations. *B*, beta coefficient; CI, confidence interval.

**Table 3:** Contribution of pathways in explaining the relation between exposure to the intervention and changes in cycle commuting time among those with lower and higher levels of active commuting at baseline.

**Path**	***B* (95%CI)**	**%effect explained**
*Stratum*: *lower active commuting at baseline. Outcome*: *increase in cycling* (*N* = *240*)		
Direct (Path 1.0)	0.27 (− 0.06, 0.59)	19.6%
Via use of path only (Path 1.1)	**1.10** (0.35, 1.85)	80.4%
**Total**	**1.37** (0.64, 2.10)	100%
		
*Stratum*: *higher active commuting at baseline. Outcome*: *increase in cycling*		
Via use of path only (Path 2.1)	**0.90** (0.35, 1.46)	N/a
		
*Stratum*: *higher active commuting at baseline. Outcome*: *decrease in cycling* (*N* = *212*)		
Via use of path only (Path 3.1)	**0.54** (0.08, 1.46)	97.2%
Via use of path, little traffic and convenient public transport (Path 3.2)	− 0.02 (− 0.03, 0.00)	− 2.2%
Via convenient public transport only (Path 3.3)	0.03 (− 0.01, 0.07)	5.1%
**Total**	**0.55** (0.10, 1.00)	100%

Bold figures are statistically significant values (*p* < 0.05); analyses were adjusted for covariates, based on full-information maximum-likelihood, with 1000 iterations. *B*, beta coefficient; CI, confidence interval.
