# Supplementary material for: Causal pathways linking environmental change with health behaviour change: Natural experimental study of new transport infrastructure and cycling to work
Source: Prev Med. 2016 Jun;87:175–82. doi: 10.1016/j.ypmed.2016.02.042 (PMC4893020; doi:10.1016/j.ypmed.2016.02.042)
Supplement: Appendix A — Pathways between exposure and change in walking on the commute [file mmc1.docx]

**Appendix A. Pathways between exposure and change in walking on the commute**

This Appendix describes the results of the analyses conducted on walking on the commute. The methods of analysing the pathways through which the introduction of the busway influences a change in walking to work were identical to the main manuscript. However, some of the measures were specific to walking.

**MEASURES
Walking on the commute**

At baseline and follow-up, participants reported all their commuting journeys and the modes of transport used over the past seven days. Participants who walked any part of their journeys to and from work were asked to report the average time walked per trip. Total weekly walking commuting time was calculated by multiplying the number of trips involving walking with the average duration of walking per trip. This measure has been shown to have acceptable criterion validity, with only a small mean overestimation compared to objectively-derived estimates.[^27^](#_ENREF_27)

Change in time spent walking was calculated by subtracting the weekly walking commuting time at baseline from the weekly walking commuting time at follow-up. This variable was skewed and was therefore categorised as either a decrease, no change (reference category), or an increase in weekly walking commuting time.

**Self-reported use**

Use of the intervention was assessed at follow-up waves in 2010, 2011 and 2012 with the item “Have you walked or cycled along any part of the footpath or cycle path beside the guided busway?”. The response categories were “Yes, I have walked beside the Busway”, “Yes, I have cycled beside the Busway” and “No, I have not walked or cycled along the paths beside the busway at all”. A dichotomous summary measure was created to indicate whether a participant had walked beside the busway between the baseline and follow-up surveys, hereafter referred to as “self-reported use”.

**RESULTS**
**Sample characteristics**The study samples used in both analytical steps are described in Table A1. A total of 414 participants were in the sample used to identify the potential pathways (step 1) and 444 participants were included in the path analyses (step 2). We did not observe any statistically significant differences either between these samples, or between either sample and the whole sample providing baseline and follow-up data.

Figure A1 shows the statistically significant results (p<0.05) of the regression analyses adjusted for covariates. The resultant model with testable pathways is shown in Figure A2

**Analysis step 1: Identification of plausible pathways**

Figure A2 shows that one pathway was identified linking the busway with an *increase in* walking (Figure A2). In this indirect pathway exposure to the busway was positively associated with use of the path for walking, which in turn was positively associated with a change in perceptions of little traffic, which was positively associated with a change in pleasantness to walk, which was positively associated with an increase in walking.

**Table A1.** Description of samples used to identify and test plausible pathways

|  | **Sample used to identify plausible pathways (N=414)** | **Sample used to test plausible pathways (N=444)** |
| --- | --- | --- |
|  | **% / mean (SD)** | **% / mean (SD)** |
| Age | 43.9 (10.8) | 44.3 (10.9) |
| Gender |  |  |
| Female | 66.4% | 65.8% |
| Male | 33.6% | 34.2% |
| Urbanicity |  |  |
| Urban | 65.9% | 66.4% |
| Rural | 34.1% | 33.6% |
| Child |  |  |
| No children | 66.9% | 67.6% |
| At least one child | 33.1% | 32.4% |
| Education |  |  |
| Lower than degree level | 25.4% | 25.5% |
| Degree level | 74.6% | 74.5% |
| Car parking at work |  |  |
| No | 30.7% | 31.1% |
| Yes, free | 37.2% | 36.9% |
| Yes, paid for | 32.1% | 32.0% |
| Home ownership |  |  |
| Does not own a home | 22.2% | 21.8% |
| Owns a home | 77.8% | 78.2% |
| Car ownership |  |  |
| Does not own a car | 12.1% | 11.7% |
| Owns at least one car | 87.9% | 88.3% |
| Baseline walking (minutes per week) | 37.3 (80.3) | 36.2 (78.9) |
| Change in weekly walking time |  |  |
| Percentage increasing | 16.9% | 16.2% |
| Minutes increased among increasers | 73.6 (68.2) | 75.1 (68.3) |
| Percentage decreasing | 18.8% | 18.7% |
| Minutes decreased among decreasers | -84.5 (72.1) | -84.6 (70.4) |

SD=Standard deviation

Four pathways linking the busway with a *decrease in* walking were identified (Figure A2 and Table A2). In the first pathway, self-reported use was positively associated with a change in the perceptions of little traffic, which was positively associated with a change in perceptions of pleasantness to walk. In turn this was negatively associated with a change in the subjective norm towards car use, which was positively associated with a decrease in walking (Path 2.1, Table A2). In the second pathway, self-reported use was positively associated with a change in the perceptions of little traffic, which was positively associated with a change in perceptions of pleasantness to walk. In turn this was negatively associated with a change in perceived behavioural control towards car use, which was positively associated with a change in subjective norm towards car use, which was positively associated with a decrease in walking (Path 2.2, Table A2). In the third pathway, proximity to the busway was positively associated with a change in perceived convenience of public transport, which was negatively associated with a change in PBC towards car use, which was positively associated with a change in subjective norm towards car use, which was positively associated with less walking (Path 2.3, Table A2). In the fourth pathway, proximity to the busway was positively associated with a change in perceived convenience of public transport, which was negatively associated with a change in attitude towards car use, which was positively associated with a change in subjective norm towards car use, which was positively associated with less walking (Path 2.4, Table A2).

**
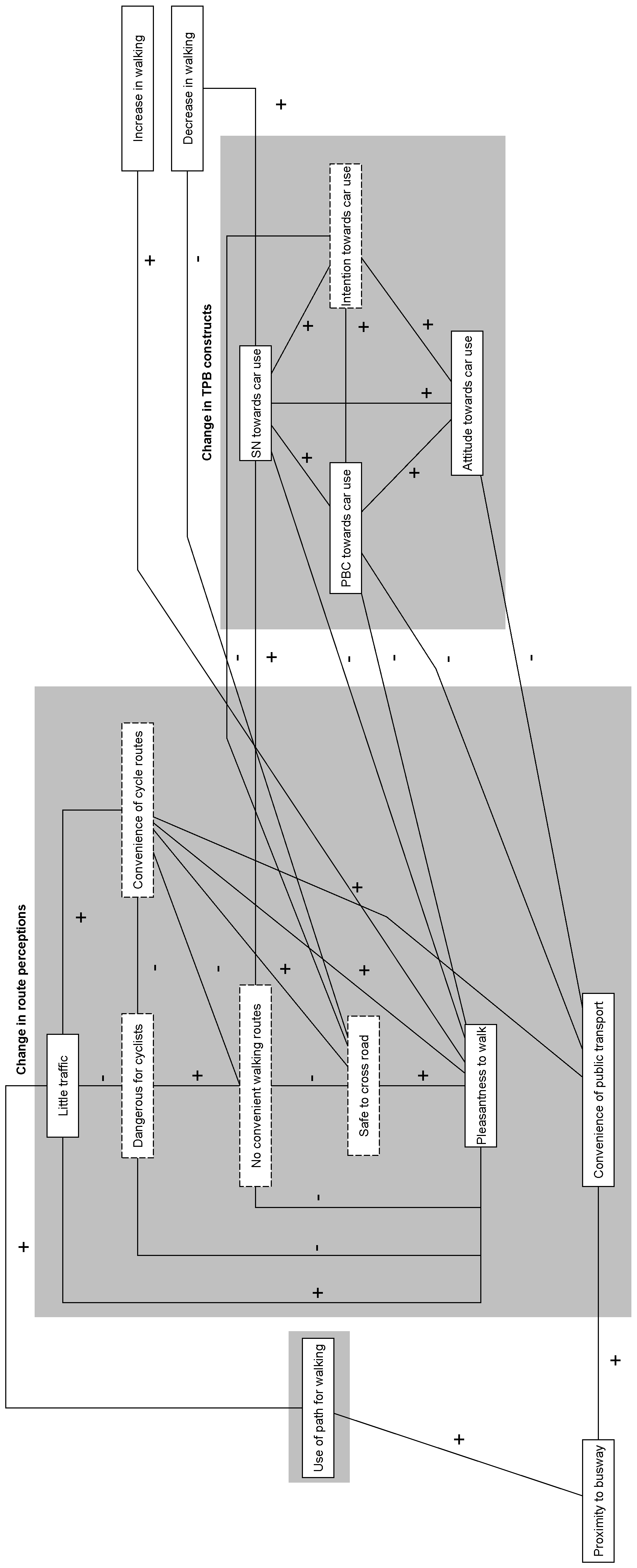

Figure A1** First refined path model linking exposure to the intervention with changes in walking commuting time. All associations shown are statistically significant (p<0.05) and either positive (+) or negative (-); dotted boxes denote potential mediators that violated the inclusion criteria for pathways to be tested (see Methods); PBC: perceived behavioural control; SN: subjective norm.

**Analysis step 2: Testing the path models**
None of these pathways was statistically significant in explaining the link between exposure to the intervention and changes in walking commuting time (Table A2).


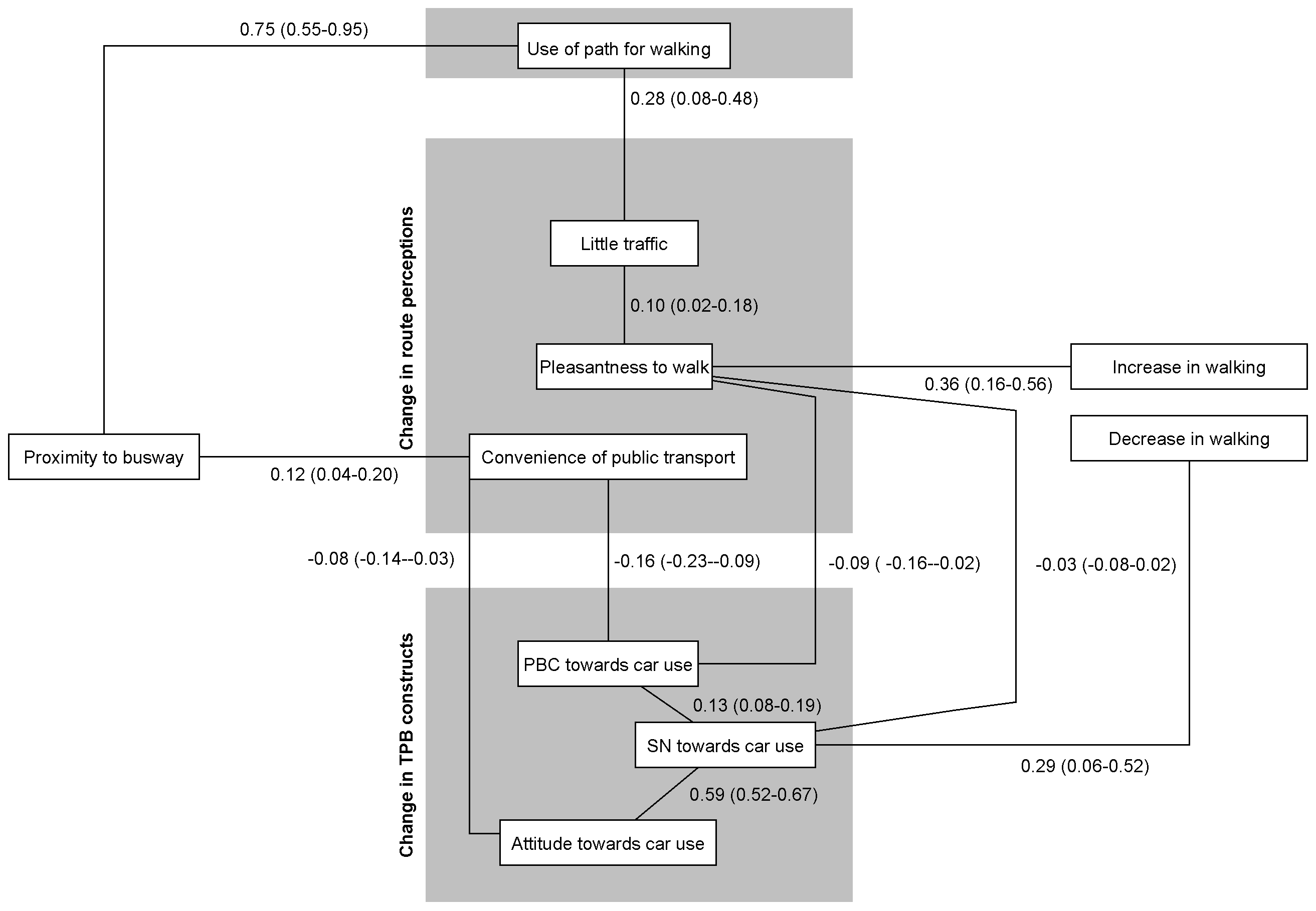


**Figure A2** Second (reduced) refined path models for whole sample. Values represent regression coefficients (95% confidence intervals) from the path analysis.

**Table A2**. Contribution of pathways in explaining the relation between exposure to the intervention and changes in walking commuting time

| **Path** | **B (95%CI)** | **%effect explained** |
| --- | --- | --- |
| *Outcome: increase in walking* |  |  |
| Via use, little traffic and pleasantness to walk (Path 1.1) | 0.008 (-0.002, 0.0017) | N/A |
| *Outcome: decrease in walking* |  |  |
| Via use, little traffic, pleasantness to walk and SN (Path 2.1) | 0.000 (-0.001, 0.000) | 7.2% |
| Via use, little traffic, pleasantness to walk, PBC and SN (Path 2.2) | 0.000 (0.000, 0.000) | 2.8% |
| Via convenience of public transport, PBC and SN (Path 2.3) | -0.001 (-0.002, 0.000) | 27.0% |
| Via convenience of public transport, attitude and SN (Path 2.4) | -0.002 (-0.004, 0.000) | 63.0% |
| **TOTAL** | -0.003 (-0.006, 0.000) | 100% |

N=444. Bold figures are statistically significant values (p<0.05); analyses were adjusted for covariates, based on full-information maximum-likelihood with 1000 iterations; B: beta coefficient, CI: confidence interval, SN: subjective norm, PBC: perceived behavioural control

In the stratified analysis for walking, three plausible pathways were identified (Figure A3). No pathways were identified for those with lower levels of active commuting at baseline. For those with higher levels of active commuting at baseline three pathways were identified (Table A3), none of which were statistically significant (Table A3).


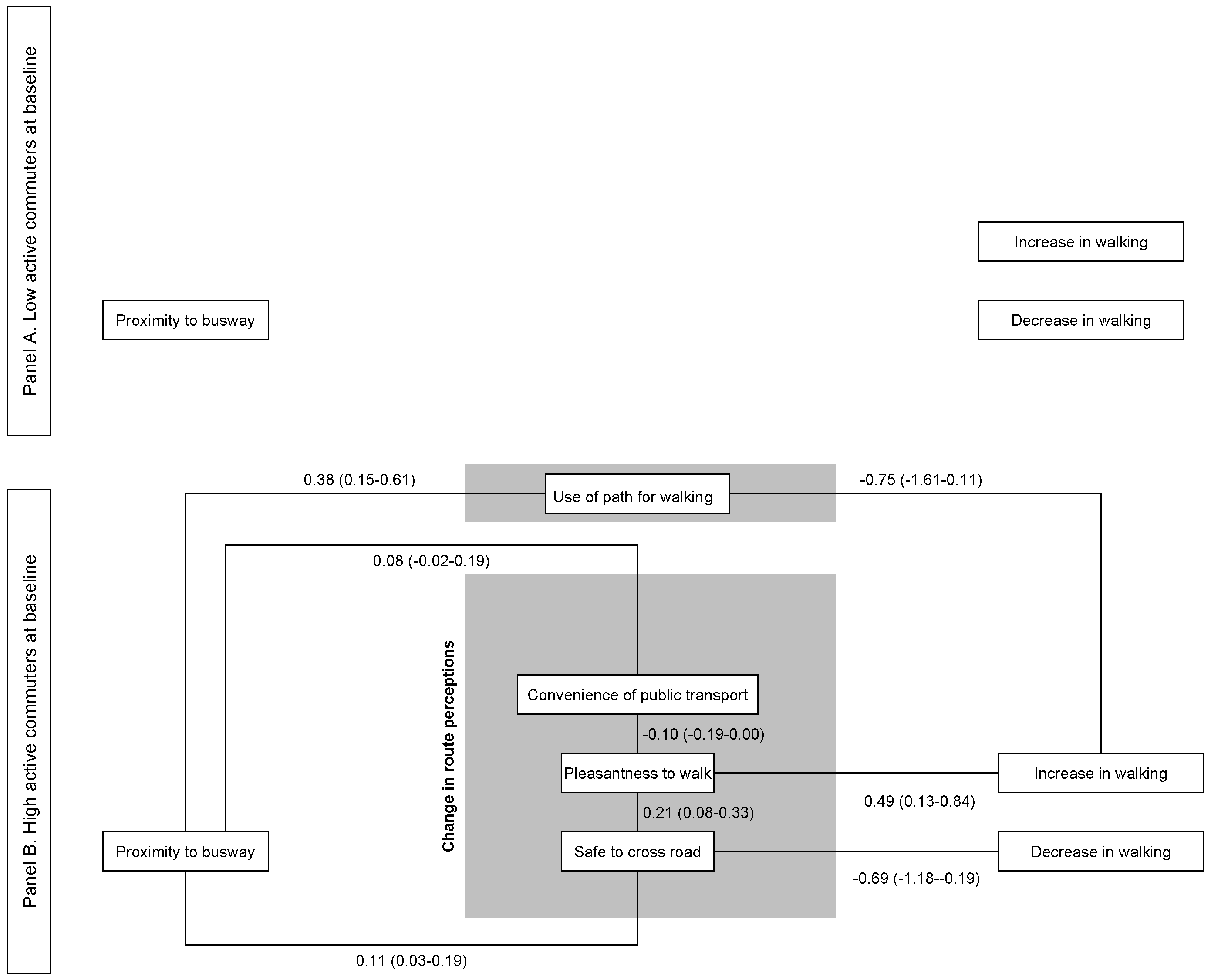


**Figure A3.** Refined path models for subsamples with lower and higher levels of active commuting at baseline. Values represent regression coefficients (95% confidence intervals) from the path analysis.

**Table A3.** Contribution of pathways in explaining the relation between exposure to the interventions and changes in walking commuting time among those with lower and higher levels of active commuting at baseline

| **Path** | **B (95%CI)** | **%effect explained** |
| --- | --- | --- |
| *Stratum: higher active commuting at baseline. Outcome: increase in walking (N=240)* |  |  |
| Via use of path only (Path 2.1) | -0.28 (-0.65, 0.08) | 103% |
| Via convenient public transport and pleasantness to walk | -0.00 (-0.01, 0.00) | 0% |
| Via safe to cross road and pleasantness to walk | 0.01 (-0.00, 0.03) | -3% |
| **TOTAL** | -0.28 (-0.64, 0.09) | 100% |
|  |  |  |
| *Stratum: higher active commuting at baseline. Outcome: decrease in walking* |  |  |
| Via safe to cross road only (Path 2.1) | -0.08 (-0.16, 0.00) | N/a |

Bold figures are statistically significant values (p<0.05); analyses were adjusted for covariates, based on full-information maximum-likelihood, with 1000 iterations; B:beta coefficient, CI:confidence interval.
