# Supplementary material for: Causal pathways linking environmental change with health behaviour change: Natural experimental study of new transport infrastructure and cycling to work
Source: Prev Med. 2016 Jun;87:175–82. doi: 10.1016/j.ypmed.2016.02.042 (PMC4893020; doi:10.1016/j.ypmed.2016.02.042)
Supplement: Appendix B — Description of mediators [file mmc2.docx]

**Appendix B. Description of mediators**

**Table B1.** Baseline and follow-up values of Theory of Planned Behaviour items, perceived environmental factors

| **Mediator/ Item** | **Valid N** | **Baseline mean (SD)** | **Follow-up mean (SD)** |
| --- | --- | --- | --- |
| **Theory of Planned Behaviour items** |  |  |  |
| *Attitude* |  |  |  |
| Overall, it would be good to use a car | 461 | 2.37 (1.46) | 2.49 (1.54) |
| It would be pleasant to use a car | 459 | 2.47 (1.28) | 2.53 (1.37) |
| *Subjective norm* |  |  |  |
| Most people who are important to me would support my using a car | 459 | 2.86 (1.36) | 2.90 (1.45) |
| Most people who are important to me think I should use a car | 459 | 2.43 (1.30) | 2.50 (1.45) |
| *Perceived Behavioural Control* |  |  |  |
| It would be easy for me to use a car | 461 | 3.10 (1.52) | 3.17 (1.58) |
| I would be able to use a car | 461 | 3.60 (1.39) | 3.54 (1.51) |
| *Intention* |  |  |  |
| I intend to use a car | 461 | 2.36 (1.60) | 2.43 (1.62) |
| I am likely to use a car | 462 | 2.48 (1.63) | 2.51 (1.66) |
|  |  |  |  |
| **Perceived environmental factors** |  |  |  |
| It is pleasant to walk | 450 | 3.53 (1.17) | 3.36 (1.20) |
| The roads are dangerous for cyclists | 462 | 3.46 (1.06) | 3.37 (1.17) |
| There is convenient public transport | 458 | 2.78 (1.29) | 2.81 (1.29) |
| There are convenient routes for cycling | 462 | 3.40 (1.18) | 3.47 (1.23) |
| There is little traffic | 461 | 1.91 (1.08) | 1.94 (1.08) |
| There are no convenient routes for walking | 459 | 2.56 (1.24) | 2.48 (1.23) |
| It is safe to cross the road | 462 | 3.32 (1.05) | 3.30 (1.06) |

All items on recoded scale from ‘strongly agree’ (5) to ‘strongly disagree’ (1); SD: standard deviation

**Table B2.** Mean and median changes of Theory of Planned Behaviour constructs and perceived environmental factors

| **Mediator/ Item** | **Mean change (SD)** | **Median (IQR) change** |
| --- | --- | --- |
| **Theory of Planned Behaviour constructs** |  |  |
| Attitude | 0.09 (0.86) | 0 (1) |
| Subjective norm | 0.06 (0.93) | 0 (1) |
| Perceived Behavioural Control | 0.00 (1.07) | 0 (1) |
| Intention | 0.05 (0.98) | 0 (0) |
|  |  |  |
| **Perceived environmental factors** |  |  |
| It is pleasant to walk | -0.16 (1.18) | 0 (1) |
| The roads are dangerous for cyclists | -0.09 (1.04) | 0 (1) |
| There is convenient public transport | 0.02 (1.19) | 0 (2) |
| There are convenient routes for cycling | 0.07 (1.16) | 0 (1) |
| There is little traffic | 0.03 (1.19) | 0 (0) |
| There are no convenient routes for walking | -0.08 (1.30) | 0 (2) |
| It is safe to cross the road | -0.03 (1.04) | 0 (1) |

SD=standard deviation, IQR=Inter-quartile range
